# The effect of intervelar veloplasty under magnification (Sommerlad’s Technique) without tympanostomy on middle ear effusion in cleft palate patients

**DOI:** 10.1186/s12887-021-02856-0

**Published:** 2021-09-01

**Authors:** Mohammad Ali Hoghoughi, Tayebeh Kazemi, Ali Khojasteh, Raha Habibagahi, Zahra Kalkate, Zeynab Zarei, Hamidreza Hosseinpour, Maryam Salimi

**Affiliations:** 1 Plastic & Reconstructive Surgery Research Center, Shiraz University of Medical Sciences, Shiraz, Iran; 2 Otolaryngology Research Center, Shiraz University of Medical Sciences, Shiraz, Iran; 3 Burn and Wound Healing Research Center, Shiraz University of Medical Science, Shiraz, Iran; 4 Orthodontic Research Center, Shiraz University of Medical Science, Shiraz, Iran; 5 Student Research Committee, Shiraz University of Medical Sciences, 71936 − 13311 Shiraz, Iran

**Keywords:** Cleft palate, Otitis media with effusion, Tympanostomy tube, Ventilation tube

## Abstract

**Objective:**

Different surgical techniques and management approaches have been introduced to manage the cleft palate (CP) and its complications, such as otitis media with effusion (OME) and auditory problems. The optimal method, as well as the ideal time for palatoplasty and ventilation tube insertion, are the subject of controversy in the literature. We aimed to evaluate The Effect of Intervelar Veloplasty under Magnification (Sommerlad’s Technique) without Tympanostomy on Middle Ear Effusion in Cleft Palate Patients.

**Methods:**

non-syndromic cleft palate patients from birth to 24 months who needed primary palatoplasty from April 2017 to 2019 were enrolled in this study. intravelar veloplasty (IVVP) surgery under magnification has been done by the same surgeon. Likewise, Otoscopy, Auditory Brainstem Response (ABR), and tympanometry were performed for all the patients before and six months after palatoplasty.

**Results:**

Tympanograms were classified into two categories according to shape and middle ear pressure, and it was done in 42 children (84 ears). Type B curve was seen in 40 cases (80 ears) before surgery which reduced significantly (*P* < 0.005) to 12 cases in the left ear and 14 cases in the right ear after surgery. So, after surgery, 70 % of the tympanogram of left ears and 66.6 % of the tympanogram of Rt ears were in normal condition (type A tympanometry). ABR was done for 43 patients (86 ears) before surgery and six months after palatoplasty. Data were shown that 40 of the patients had mild to moderate hearing loss before surgery, which reduced significantly (*P* < 0.005) to 9 in the left ear and 11 in the right ear after palatoplasty. So, after surgery, 79 % of ABR of left ears and 73.8 % of ABR of right ears were in normal status (normal hearing threshold).

**Conclusions:**

Intervelar veloplasty under magnification (Sommerlad’s technique) significantly improved the middle ear effusion without the need for tympanostomy tube insertion.

## Introduction

The orofacial cleft with the prevalence of one in 700 births is considered the most common birth anomaly [1]. Likewise, persistent eustachian tube dysfunction is thought to be the primary factor responsible for the higher rates of more serious middle ear pathology observed in children with cleft palate, such as tympanic membrane perforation, middle ear atelectasis, cholesteatoma, and otitis media with effusion (OME) [2]. Moreover, OME entangles approximately 30 to 40 % of children at least once while rising to more than 90 % in children with CP [3, 4].

Insertion of ventilation tubes has been considered as the optimal treatment for otitis media in patients with cleft palate, even though controversy surrounds the timing of ear tube placement. Furthermore, concerning excessive compliance with the eustachian tube, high rates of otorrhea following myringotomy and ventilation tube insertion before palatoplasty have been reported [5].

It was also shown that chronic otorrhea stopped only after palatoplasty in a few children who had early myringotomy and tube insertion [6, 7]. Likewise, no long-term research has been conducted to evaluate the effect of early placement of ventilation tubes on speech outcomes in patients born with cleft palate. Therefore, various surgical procedures have been introduced to correct the anatomic structures of patients with cleft palate along eliminate the problem.

Among different surgical techniques, the Sommerlad and Furlow palatoplasty techniques seemed to generate the best outcomes for middle ear function and speech. The intravelar veloplasty (IVVP), which was planned by Kriens and propagated by Sommerlad, based on the anteriorly malpositioned bundles dissection from the posterior edge of the hard palate followed by repositioning levator sling and palatal musculature in the midline along with radical muscle dissection as well as posterior repositioning performed under the operating microscope [8].

This paper aims to show the outcome of intravelar veloplasty (IVVP) surgery under magnification (Sommerlad’s technique) on eustachian tube function and the middle ear status.

## Materials and methods

### Study design and patient selection

In this cross-sectional study, non-syndromic cleft palate patients from birth till 24 months needed primary palatoplasty, referring to the Cleft Lip and Palate Center of Shiraz University of Medical Sciences from April 201 to April 2019, were enrolled in this study. Patients who had anomalies of the external ear that probe insertion was not possible and had sensorineural hearing loss were excluded from the study. Likewise, the patients who suffered from extremely severe middle ear effusion under the guide of otorhinolaryngologists were referred for tympanostomy tube insertion and were excluded from our study. Intravelar veloplasty (IVVP) surgery under magnification has been done by the same surgeon for all the involved patients.

### Surgical technique

**Fig. 1:**
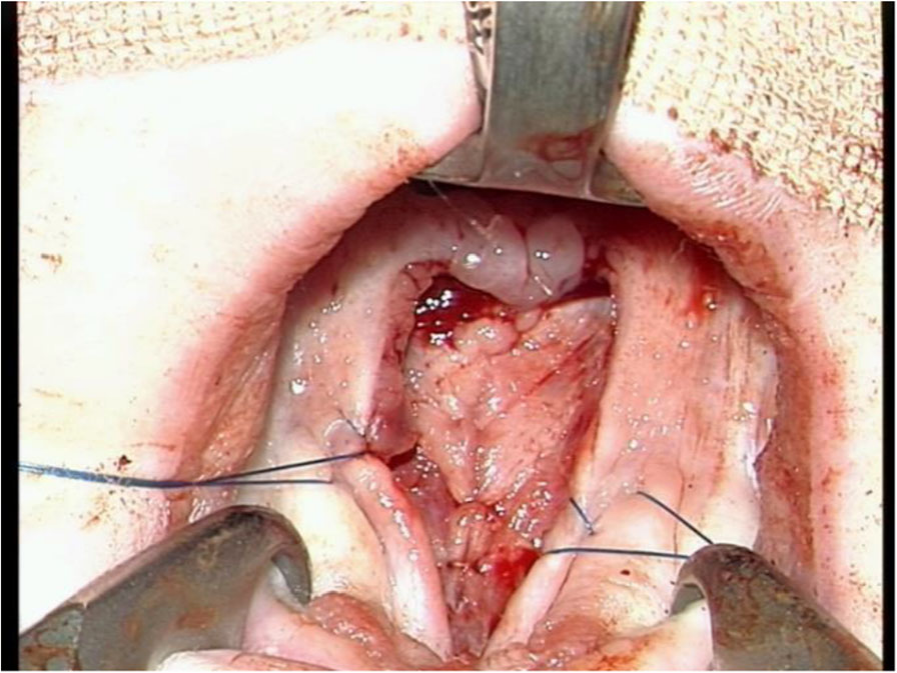
Nasal layer from uvula till the hard palate was closed with 5.0 PDS

Under general anesthesia in the supine position, the head was hyperextended, and a rolled towel was inserted under the shoulder. Then, we applied a mouth gag retractor for exposure. After that, lidocaine and epinephrine (1/200,000) were injected into the hard and soft palate and waited for 7–10 min. Intervellar veloplasty (IVVP) was started with an incision between the oral and nasal layer, and we could find this junction under magnification (× 2–3, VARIO,700 Zeiss). The incision was extended enough to the posterior part of the hard palate edges properly. The oral layer was elevated from muscles and nasal layers. At this stage, the nasal layer from the uvula to the hard palate was closed with 5.0 Polydioxanone (PDS) (Fig. 1).

**Fig. 2:**
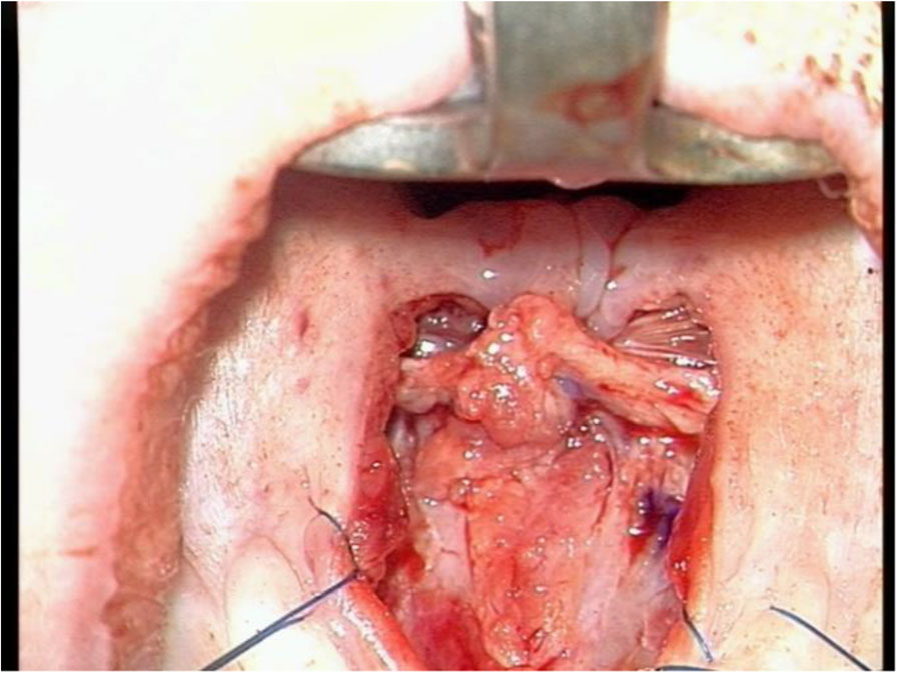
Reposition of levator muscles to the most posterior part of the hard palate

At this important stage, levator velli palatine and a small part of tensor velli palatine muscles were detached under magnification (×4–5) from the nasal layer precisely and repositioned to the most posterior part of the soft palate (Fig. 2). In the end, the oral layer was closed with 4.0 PDS. If any tension of midline closure is present, the relaxing incision would be done laterally.

### Pre and postoperative assessment

Auditory Brainstem Response (ABR) is an electrophysiologic assessment tool for estimating threshold, for newborn hearing screening, or for determining whether a hearing loss may be sensory, neural, or retrocochlear and was performed for all the patients before and 6 months after palatoplasty. Likewise, otoscopy examination also was accomplished for all of them. The Auditory Brainstem Response (ABR) threshold detection test was performed by click stimuli from 0 to 100 dB HL in the frequency 1000 to 4000 HZ. The hearing assessment was done by ABR using Eclipse from Intracoustic Company. The mean hearing loss was calculated for each ear as an average of hearing levels at 0-100 dB. The ears were classified into seven hearing level groups according to Clark classification [9], -10 -15 dB was considered normal hearing, 16–25 dB was considered a slight hearing loss, 26–40 dB was considered a mild hearing loss, 41–55 dB was considered moderate, 56–70 dB was considered moderately severe,71–90 dB was considered severe and above 90 dB was considered profound hearing loss.

Tympanometry, which can be used to evaluate the middle ear function, was done in all children before and six months after palatoplasty to assess the middle ear using AZ 26 tympanometer from intracystic company tympanometer with a probe frequency of 226 Hz. Tympanograms are categorized according to the shape of the plot and the tympanometric findings were classified according to Jerger as a normal tympanogram which is labeled Type A, that represent a normal pressure in the middle ear with normal mobility of the eardrum and ossicles and middle-ear pressure between + 50 and − 99 mmH^sub 2^O; Type B for flat trace without a well-defined compliance peak, and C tympanograms for middle-ear pressure between − 100 to -400[M1] mm-H^sub 2^O.7, Type B tympanogram may reveal fluid in the middle ear, perforation of the tympanic membrane, scarring of the tympanic membrane, lack of contact between the ossicles, or a tumor in the middle ear [10].

### Ethical consideration and statistical analysis

The written informed consent was signed by the parents of the patients and the protocol of the study was approved by the local ethical committee of Shiraz University of Medical Sciences (IR.SUMS.REC.1398.535). Descriptions were given to parents about how to do the test and how to respond to the examiner. Individuals with full satisfaction participate in this research, and written consent will be signed by one of the parents.

### Statistical analysis

After data collection, the information was entered into version 26 of SPSS software for statistical analysis. We used the paired T-test to analyze the tympanometric data of the patients before and after the palatoplasty. The frequencies of types B, C, and A were calculated for the total sample and the bilateral cleft lip and palate (BCLP), unilateral cleft lip and palate (UCLP), and isolated cleft palate (ICP) groups. We also merged the two types A and B for statistical results.

## Results

A total of 43 patients (16 male and 27 Female) with a mean age of 12 months were included in the study. Among them, 21 patients had ICP, 17 had UCLP, 3 had BLCLP, and two had submucous clefts. The first table contains the demographic data (Table 1).

**Table 1:** Demographic data of cleft palate patients and their correlation with age

***Variable***	***Average± SD***^a^***/ frequency***	***Gender***		
		***Male***	***Female***	***P. value***
***Age (month)***	11.5 ± 2.1	11.4 ± 2.3	11.6 ± 2.0	0.703
***ICP***^b^	21	8	13	0.909
***UCLP***^c^	17	6	11	0.838
***BLCLP***^d^	3	2	1	0.285
***Submucosa cleft***	2	0	2	0.276

^a^Standard deviation

^b^Isolated cleft palate

^c^Unilateral cleft lip and palate

^d^Bilateral cleft lip and palate

Tympanogram results were divided into two categories (A and B) according to shape and middle ear pressure, and it was done in 42 children (84 ears). Type B curve was seen in 40 cases before surgery which reduced to 12 cases in the left ear and 14 cases in the right ear after surgery. So, after surgery, 70 % of the tympanogram of left ears and 66.6 % of the tympanogram of right ears were in normal condition (type A tympanometry). A comparison of Tympanogram before and post-surgery in our study revealed statistically significant differences at this test (*P* < 0.005).

ABR was done for 43 patients (86 ears) before surgery and 6 months after palatoplasty. Data were shown that 40 of the patients had mild to moderate hearing loss before surgery which reduced to 9 in the left ear and 11 in the right ear after palatoplasty. So, after surgery, 79 % of left ABR and 73.8 % of ABR of right ears were in normal status (normal hearing threshold). The difference between the two groups regarding ABR in the right ear and left ear was statistically significant (*P* < 0.005).

Table 2 demonstrates the details of ABR and tympanogram before and 6 months after surgery.

**Table 2:** Assessment of ABR and tympanogram before and 6 months after surgery

Variable	Pre-operation Number (%)	Post-operation Number (%)	*P*. value
Abnormal ABR^a^			
Right	40 (95.2)	11 (25.6)	*P* < 0.005
Left	40 (95.2)	9 (20.9)	
Abnormal Tym. ^b^			
Right	40 (93.0)	14 (33.3)	*P* < 0.005
Left	40 (93.0)	12 (28.6)	

^a^*ABR* Auditory Brainstem Response

^b^*Tym* Tympanogram

About 30 % of the 93 % of the population who experienced moderate to severe otitis media along with effusion recovered and became normal after palatoplasty surgery which was statistically significant. Likewise, 66.67 % of the patients had mild to moderate otitis media and effusion before surgery which reduced to 33.33 % after palatoplasty.

## Discussion

CP is not an isolated problem and is accompanied by many disorders such as auditory problems as well as middle ear effusion. The goals for treatment of the cleft palate patient besides normal speech, maxillofacial growth, and avoiding fistulas are eliminating the problems of middle ear infections as well as improve the hearing ability of these patients. The optimal management and intervention time of CP and each of its complications has been widely discussed and were the subject of controversy in the literature. Likewise, eustachian tube dysfunction which is considered as one of the major problems in cleft palate patients must have managed accurately not only to prevent the future OME but also for proper hearing and following speech ability. The results of our study indicated the improvement in middle ear function and hearing conditions as well as reducing the need for ventilation tube insertion in cleft patients after IVVP Under magnification.

Based on our results, the OME reduced from 95 to 32 %, 6 months after Sommerlad IVVP. It shows that the applied method performed significantly in the improvement of the middle ear effusion. Several studies approved the effect of palatoplasty in reducing the incidence of OME, while they did not find any statistically significant correlation between the surgical technique and the reducing impact on middle ear effusion [11–14]. On the other hand, Hassan et al. reported that Sommerlad IVVP was associated considerably with a lower incidence of OME in comparison with the (Veau-Wardill-Kilner) VWK palatoplasty [15]. Overall with respect to the results of current and previous studies.

According to the hearing assessments by ABR, 93 % had mild to moderate hearing loss before surgery which reduced significantly to 23 %, 6 months after Sommerlad IVVP. Antonelli et al. Carrol et al. reported the superiority effect of Furlow palatoplasty on von Langenbeck, 2-flap, and the VWK palatoplasties. Likewise, Musgrave et al. found the better result of hearing ability in von Langenbeck in comparison with VWK palatoplasty, while surgical technique was not a related factor to hearing tech auditory function in a study that was done by Lithovius et al. It is noteworthy that the study above did not include the Sommerlad IVVP technique. Therefore, a comprehensive comparison between different surgical techniques regarding the post-operation auditory function has not been possible so far, but it can be said that all palatal reconstruction methods improve hearing function.

There have been different reports in terms of the ideal time for management of OME via tympanostomy tube. Based on our data from tympanogram assessments which have been done before surgery and 6 months after surgery, a significant amount of OME was revealed 6 months after palatoplasty without the need for a tympanostomy tube. According to the parallel study, Hassan et al. believed that secretory otitis media improved 6 months after palatoplasty without the requisite ventilation tube insertion. He also reported that IVVP is a contributing factor in eustachian tube normalization function [15]. Similarly, other studies corroborated that palatal muscle reconstruction and relocation decrease the rate of eustachian tube dysfunction [16, 17]. Brgoch et al. and D’Andréa et al. emphasized the role of Sommerlad IVVP in reducing the incidence need of ventilation tube placement [11, 18].

On the contrary, Mantilla et al. suggested the combination of palatoplasty and ventilation tube insertion. He pointed to the potential noxious effect of repeated use of anesthetics on neurocognitive development while he did not find any significant correlation. The author also cited the association of language development with more cleft palate-related surgery. The conclusion mentioned above could be wrong since cleft palate affects speech development and patients with the more severe form of deformity logistically need more surgery. Therefore, the lower speech-related abilities were attributed to the more disease severity [19]. Likewise, Balraj et al. suggested that combining procedures may be more cost-effective and decrease the total burden on families [20].

Furthermore, in vitro evidence showed the apoptosis effect of anesthesia exposure on the nervous system in rodents [21]. On the other hand, several clinical studies denied the deleterious neurodevelopmental effect of anesthetics in pediatrics [22, 23]. To clarify the above statements, it seems that equal consideration of the number of surgeries in both combined and separate groups of surgery caused a mistake during comparing. To describe more precisely, palatoplasty and palatal muscles reconstruction decrease the recurrent incidence of OME and so the total number of tympanostomy tube insertions was reduced after 6 months of the first operation. Therefore, the total amount of anesthetic exposure, hospital stay, and the burden is expected to reduce theoretically.

It also should be considered that simultaneous procedures require collaboration and the presence of two different specialized teams. For the same reason, an uninvited interruption, suspension, and delays in scheduling the procedures are unavoidable.

From the point of view of surgical complications, ventilation tube insertion has been proven to associate with tympanic membrane perforation, otorrhea, and tympanosclerosis. The cumulative evidence at the present study indicates that the aforementioned complications will be decreased by improving the eustachian tube function by the IVVP under magnification (Sommerlad technique) and post-operation follow-up in 6 months.

Our perusal was not without limitation. The observational methodology does not allow us to demonstrate the efficacy of the used approach properly. Moreover, the possibility of selection bias should also be addressed. Likewise, the control group of combined surgery did not exist. The absence of the control group was due to ethical considerations. Since the aforementioned approach and the surgical method had the best result for the patients clinically according to the surgeon’s opinion of this center, it was not possible to have a control group who underwent surgery in another way according to the ethical consideration. Furthermore, we did not include the patients with simultaneous cleft palate, and craniofacial syndromes considered the contributing factor of recurrent OME.

## Conclusions

Intervelar veloplasty under magnification significantly improved the middle ear effusion for 63 % and also hearing function for 70 % without the need for simultaneous tympanostomy tube insertion during the 6 months after surgery. Therefore, tympanostomy tube insertion may not be necessary, at least after this technique of primary palatoplasty. However, regular follow-up is needed to evaluate the status of middle ear effusion.

## Data Availability

SPSS data of the participant can be requested from the authors. Please write to the corresponding author if you are interested in such data.
